# Author Correction: Machine learning-based analytics of the impact of the Covid-19 pandemic on alcohol consumption habit changes among United States healthcare workers

**DOI:** 10.1038/s41598-023-34081-3

**Published:** 2023-04-26

**Authors:** Mostafa Rezapour, Muhammad Khalid Khan Niazi, Metin Nafi Gurcan

**Affiliations:** Center for Biomedical Informatics, Wake Forest University School of Medicine, Winston-Salem, NC USA

Correction to: *Scientific Reports* 10.1038/s41598-023-33222-y, published online 12 April 2023

The original version of this Article contained an error in the Acknowledgements section.

“We would like to thank John D. Ford for his assistance during his internship in the Center for Biomedical Informatics at Wake Forest University School of Medicine.”

now reads:

“The project described was supported in part by UL1 TR001420 (PI: McClain) from National Center for Advancing Translational Sciences. The content is solely the responsibility of the authors and does not necessarily represent the official views of the National Center for Advancing Translational Sciences or the National Institutes of Health. Furthermore, we would like to thank John D. Ford for his assistance during his internship in the Center for Biomedical Informatics at Wake Forest University School of Medicine.”

The original Article has been corrected.

